# Supplementary figures and images for: Microbiome Interaction Networks and Community Structure From Laboratory-Reared and Field-Collected Aedes aegypti, Aedes albopictus, and Culex quinquefasciatus Mosquito Vectors
Source: Front Microbiol. 2018 Sep 10;9:2160. doi: 10.3389/fmicb.2018.02160 (PMC6140713; doi:10.3389/fmicb.2018.02160)

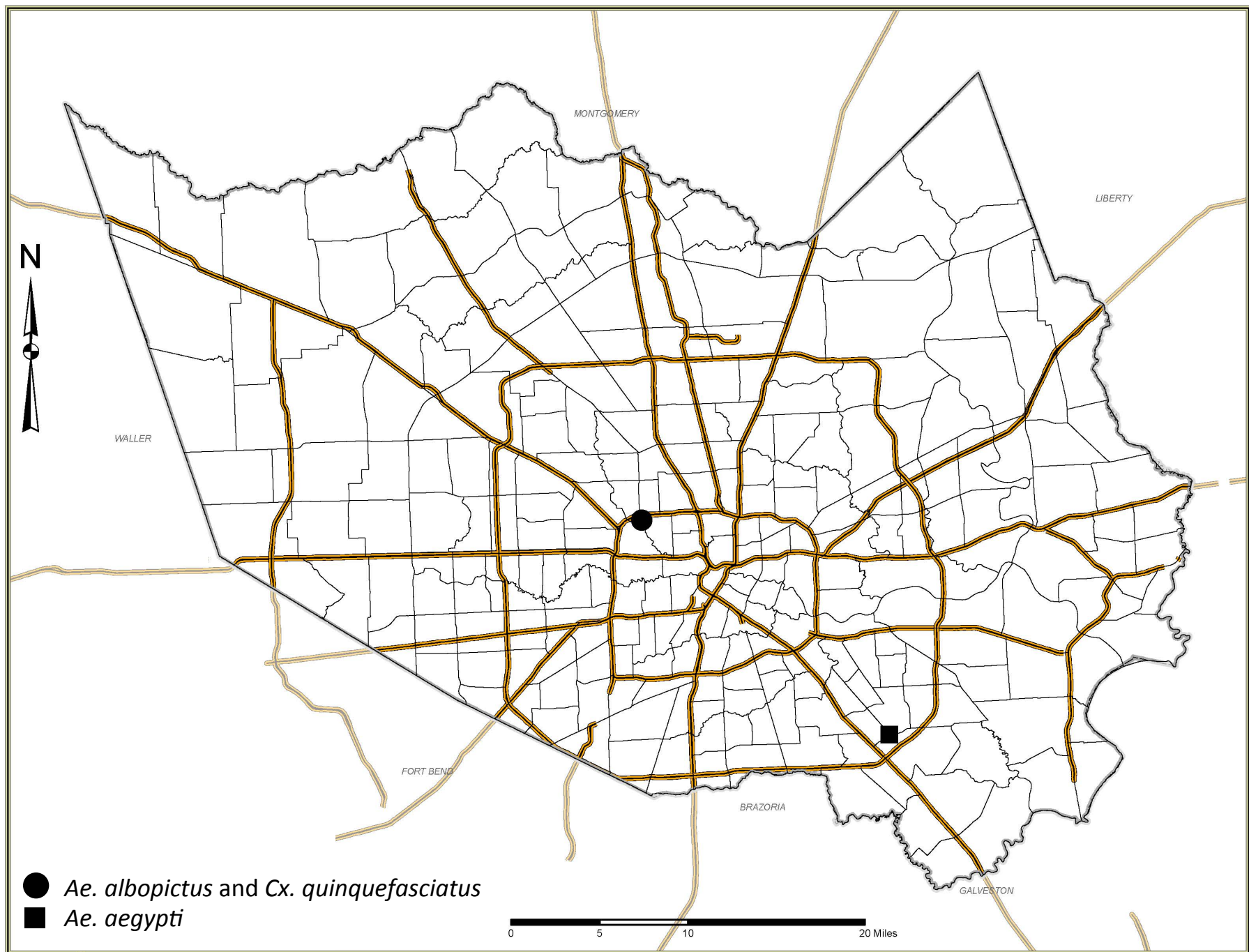

Supplement: FIGURE S1 — Map of Houston, Texas, indicating the field collection sites. [file Image_1.PDF]

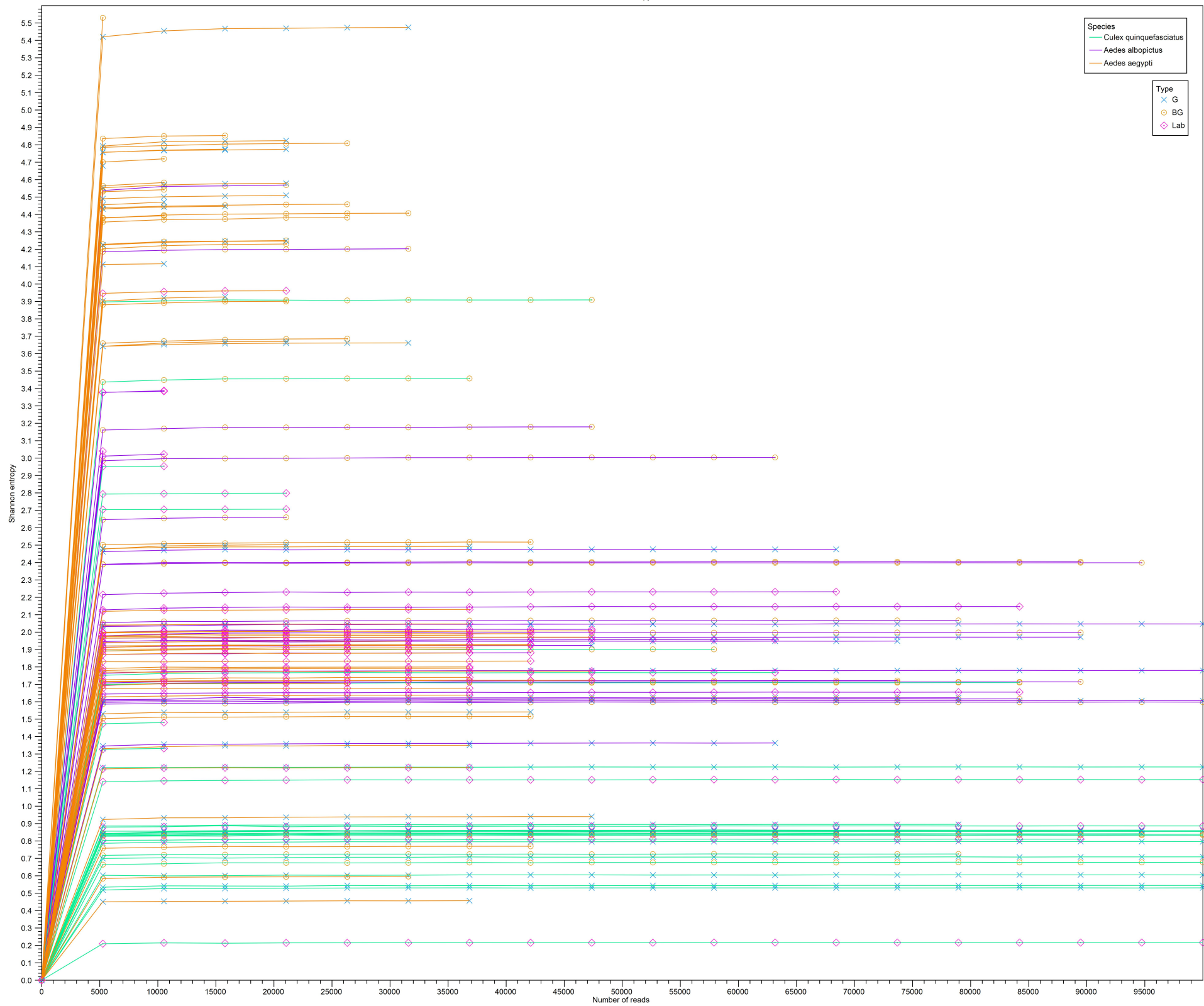

Supplement: FIGURE S2 — Shannon entropy rarefied at intervals between 0 and 100,000 reads in each sample from different groups (G, BG, Laboratory) in all three mosquito species. [file Image_2.PDF]

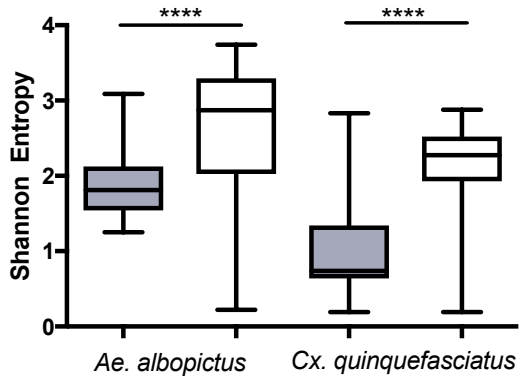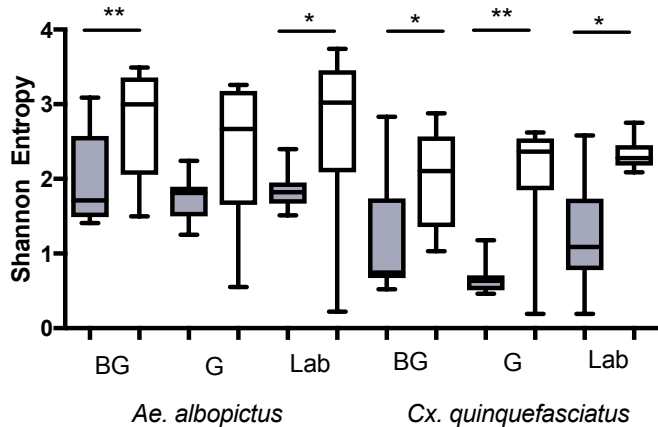

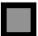 *Wolbachia* present

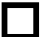 *Wolbachia* computationally removed

Supplement: FIGURE S4 — Shannon diversity of Ae. albopictus and Cx. quinquefasciatus with and without Wolbachia. For analysis of samples without the endosymbiont, Wolbachia reads were computationally excluded from the analysis and then Shannon diversity was recalculated (∗P < 0.05, ∗∗P < 0.01, ∗∗∗∗P < 0.0001). [file Image_4.PDF]

## *Serratia*

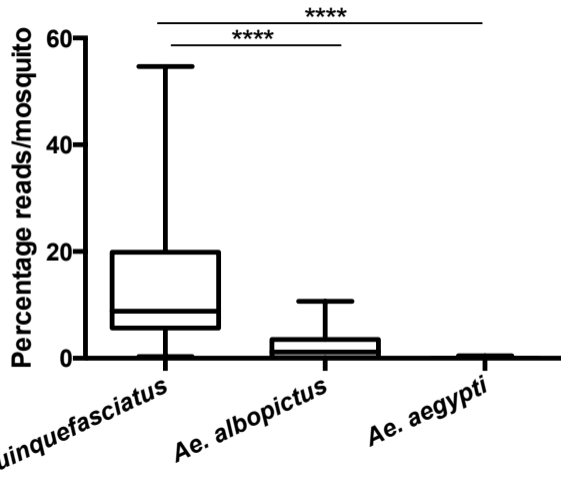

## *Aeromonas*

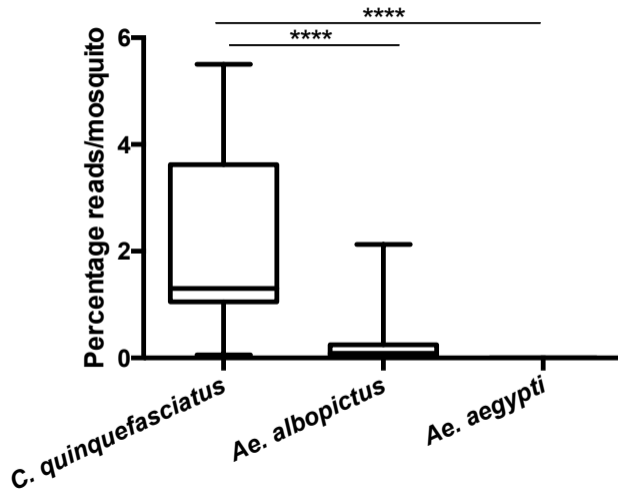

Supplement: FIGURE S5 — Relative abundance of Serratia and Aeromonas from high-throughput sequencing in Cx. quinquefasciatus, Ae. albopictus and Ae. aegypti mosquitoes reared in the lab. Data were analyzed using a one-way ANOVA using Tukeys method for pairwise comparisons (∗∗∗∗P < 0.0001). [file Image_5.PDF]
