# Supplementary material for: Microbiome Interaction Networks and Community Structure From Laboratory-Reared and Field-Collected Aedes aegypti, Aedes albopictus, and Culex quinquefasciatus Mosquito Vectors
Source: Front Microbiol. 2018 Sep 10;9:2160. doi: 10.3389/fmicb.2018.02160 (PMC6140713; doi:10.3389/fmicb.2018.02160)
Supplement: FIGURE S3 — Heat maps indicating bacterial relative abundance for the three mosquito species. OTUs were grouped to genus level or higher ranks (when genus was ambiguous) and the relative abundance indicated by color for each individual (column) is shown. The upper heat map is with Wolbachia present while the lower has Wolbachia excluded. The dendrogram/clustering of the bacteria is generated based on their relative abundance correlation across samples. [file Image_3.PDF]

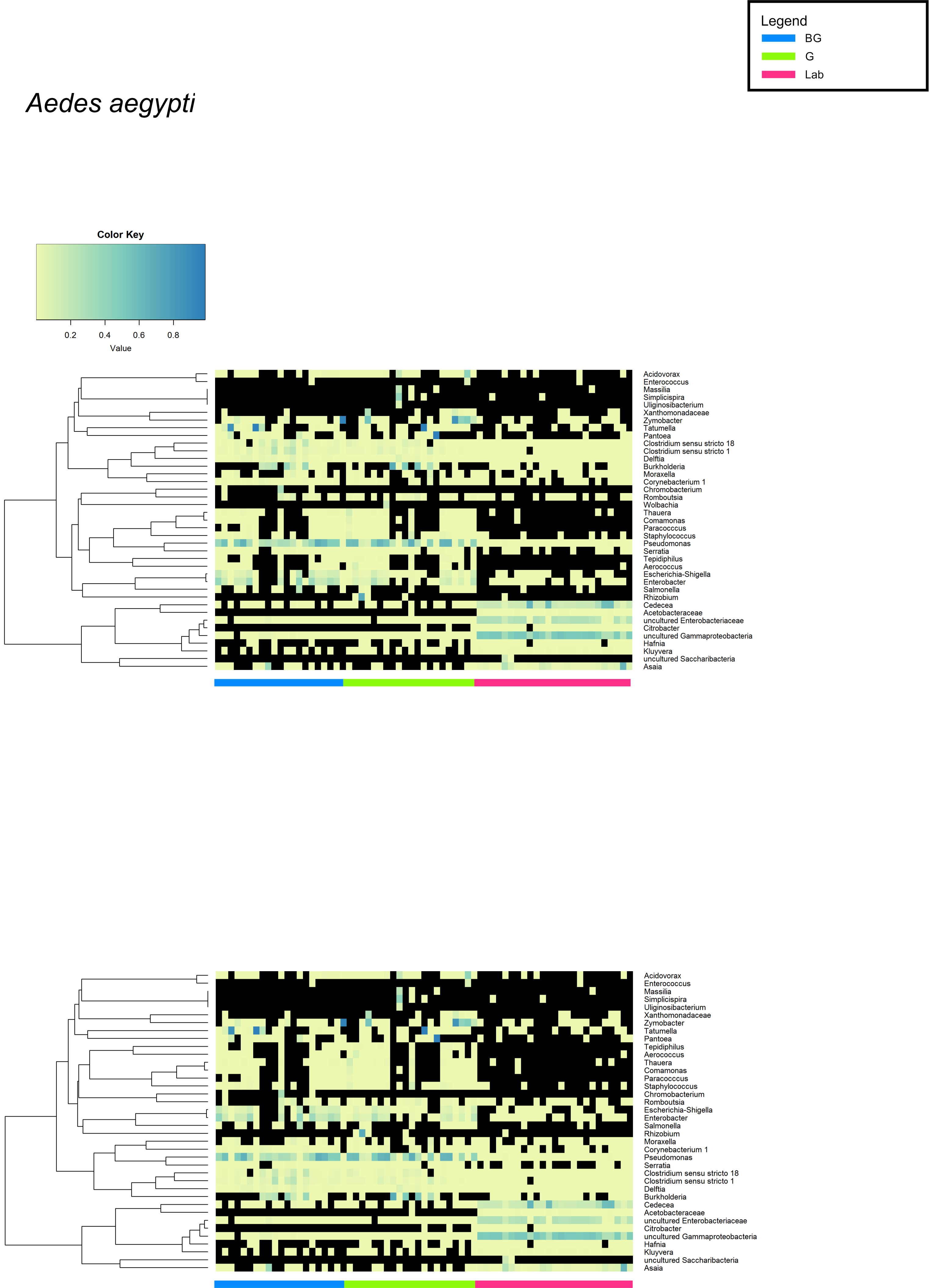

Legend
BG
G
Lab
Aedes aegypti
Color Key
0.2
0.4
0.6
0.8
Value
Acidovorax
Enterococcus
Massilia
Simplicispira
Uliginosibacterium
Xanthomonadaceae
Zymobacter
Tatumella
Pantoea
Clostridium sensu stricto 18
Clostridium sensu stricto 1
Delftia
Burkholderia
Moraxella
Corynebacterium 1
Chromobacterium
Romboutsia
Wolbachia
Thauera
Comamonas
Paracocccus
Staphylococcus
Pseudomonas
Serratia
Tepidiphilus
Aerococcus
Escherichia-Shigella
Enterobacter
Salmonella
Rhizobium
Cedecea
Acetobacteraceae
uncultured Enterobacteriaceae
Citrobacter
uncultured Gammaproteobacteria
Hafnia
Kluyvera
uncultured Saccharibacteria
Asaia
Acidovorax
Enterococcus
Massilia
Simplicispira
Uliginosibacterium
Xanthomonadaceae
Zymobacter
Tatumella
Pantoea
Tepidiphilus
Aerococcus
Thauera
Comamonas
Paracocccus
Staphylococcus
Chromobacterium
Romboutsia
Escherichia-Shigella
Enterobacter
Salmonella
Rhizobium
Moraxella
Corynebacterium 1
Pseudomonas
Serratia
Clostridium sensu stricto 18
Clostridium sensu stricto 1
Delftia
Burkholderia
Cedecea
Acetobacteraceae
uncultured Enterobacteriaceae
Citrobacter
uncultured Gammaproteobacteria
Hafnia
Kluyvera
uncultured Saccharibacteria
Asaia

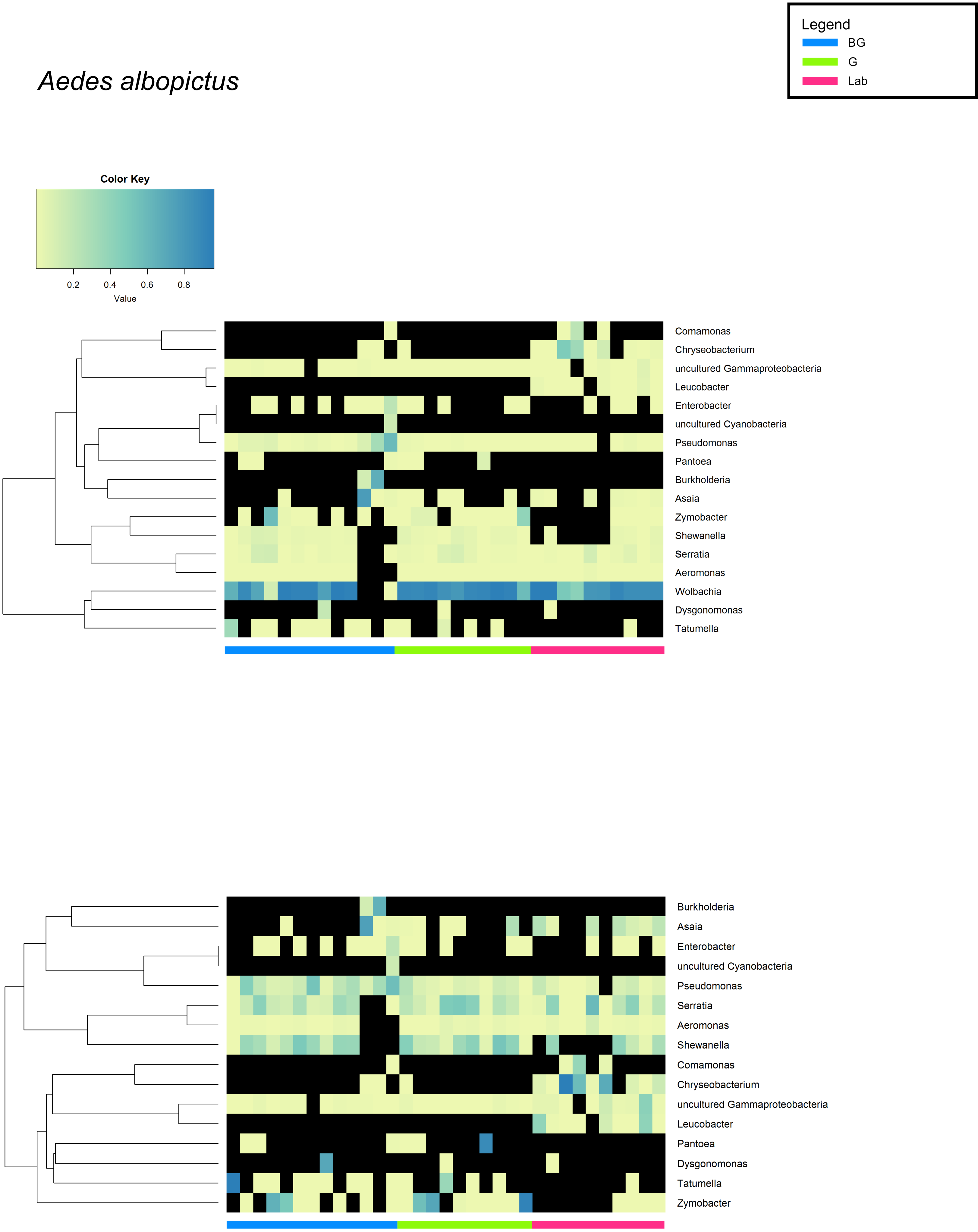
Aedes albopictus
Legend
BG
G
Lab
Color Key
0.2
0.4
0.6
0.8
Value
Comamonas
Chryseobacterium
uncultured Gammaproteobacteria
Leucobacter
Enterobacter
uncultured Cyanobacteria
Pseudomonas
Pantoea
Burkholderia
Asaia
Zymobacter
Shewanella
Serratia
Aeromonas
Wolbachia
Dysgonomonas
Tatumella
Burkholderia
Asaia
Enterobacter
uncultured Cyanobacteria
Pseudomonas
Serratia
Aeromonas
Shewanella
Comamonas
Chryseobacterium
uncultured Gammaproteobacteria
Leucobacter
Pantoea
Dysgonomonas
Tatumella
Zymobacter

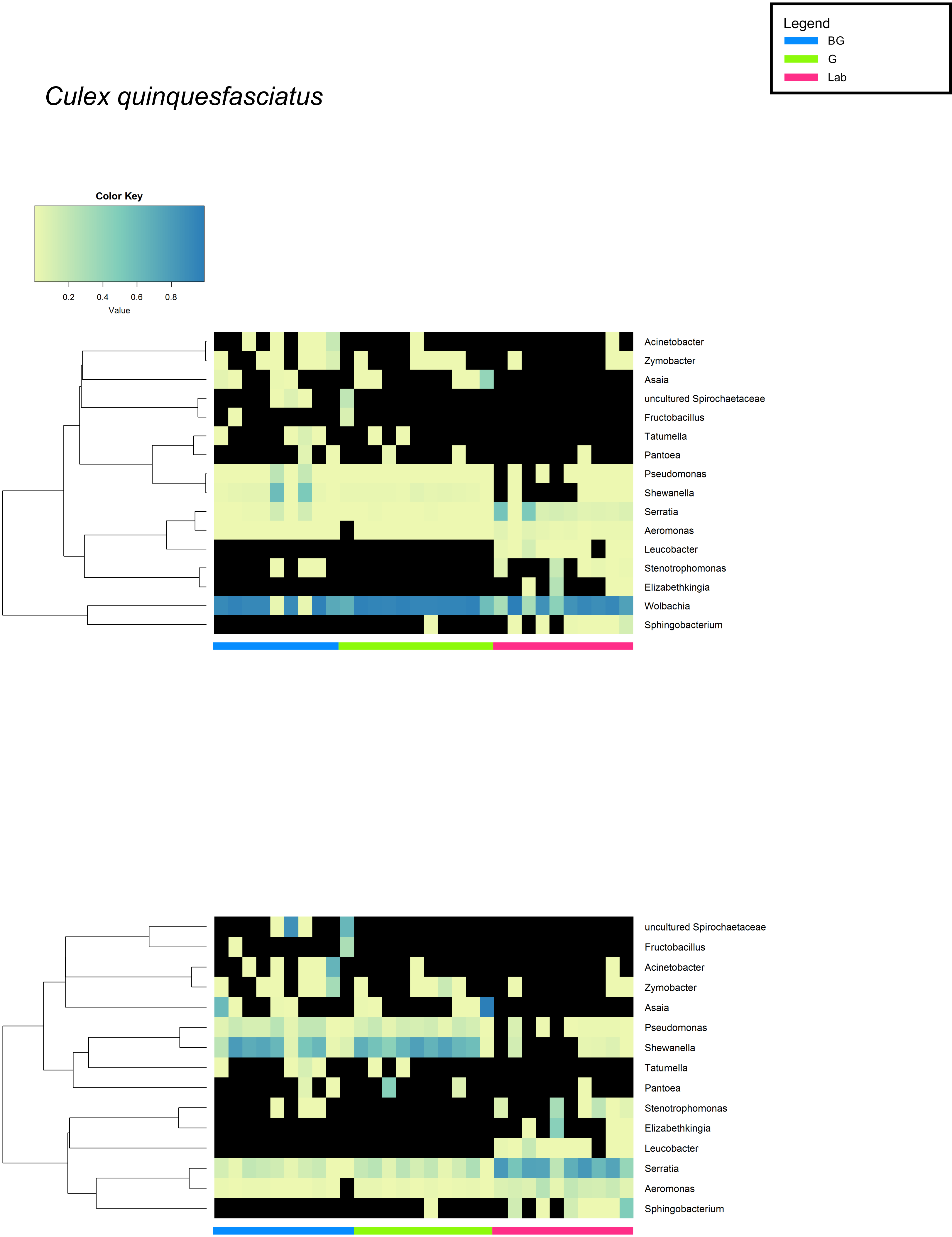

Culex quinquesfasciatus
Legend
BG
G
Lab
Color Key
0.2
0.4
0.6
0.8
Value
Acinetobacter
Zymobacter
Asaia
uncultured Spirochaetaceae
Fructobacillus
Tatumella
Pantoea
Pseudomonas
Shewanella
Serratia
Aeromonas
Leucobacter
Stenotrophomonas
Elizabethkingia
Wolbachia
Sphingobacterium
uncultured Spirochaetaceae
Fructobacillus
Acinetobacter
Zymobacter
Asaia
Pseudomonas
Shewanella
Tatumella
Pantoea
Stenotrophomonas
Elizabethkingia
Leucobacter
Serratia
Aeromonas
Sphingobacterium

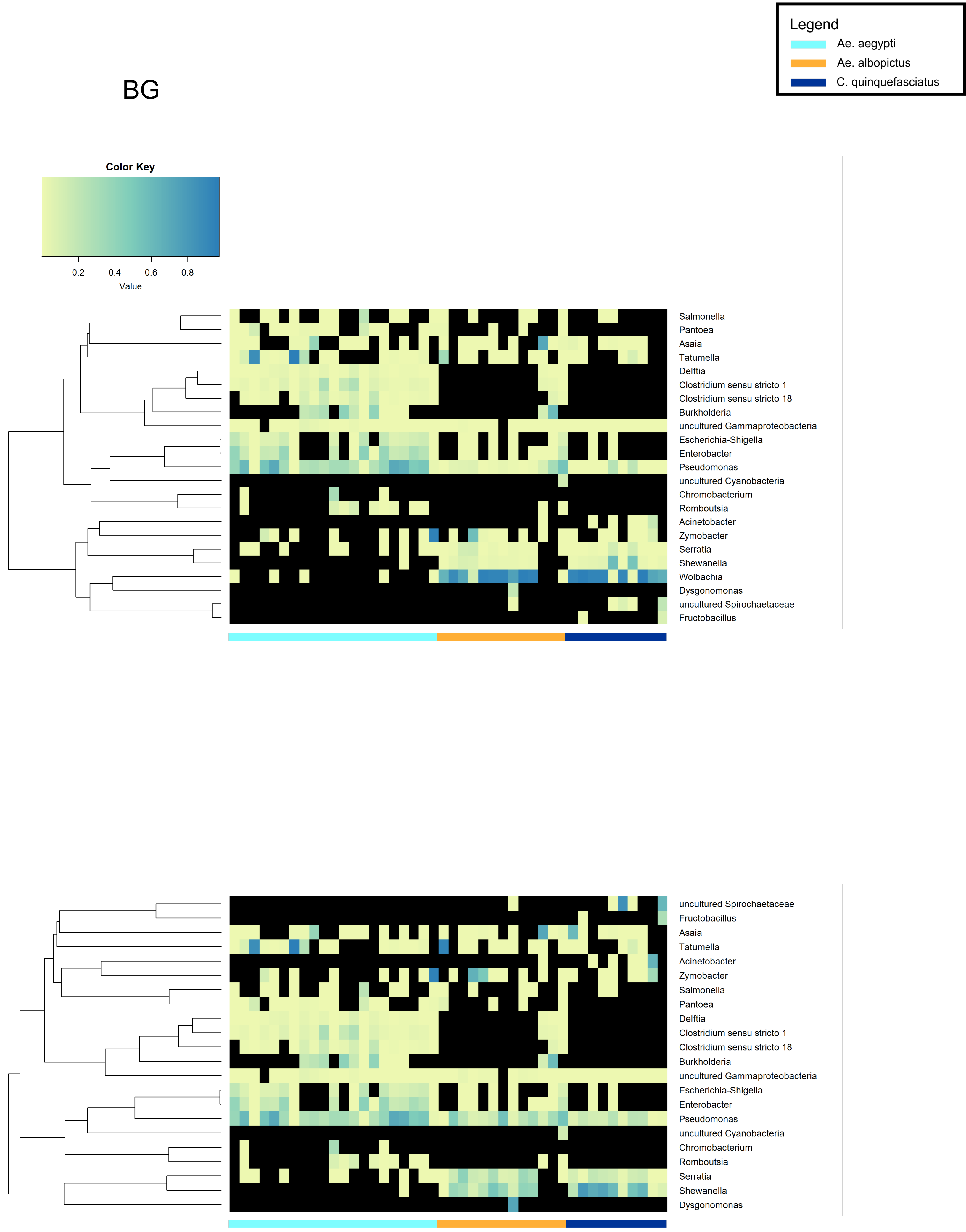
BG
Legend
Ae. aegypti
Ae. albopictus
C. quinquefasciatus
Color Key
0.2
0.4
0.6
0.8
Value
Salmonella
Pantoea
Asaia
Tatumella
Delftia
Clostridium sensu stricto 1
Clostridium sensu stricto 18
Burkholderia
uncultured Gammaproteobacteria
Escherichia-Shigella
Enterobacter
Pseudomonas
uncultured Cyanobacteria
Chromobacterium
Romboutsia
Acinetobacter
Zymobacter
Serratia
Shewanella
Wolbachia
Dysgonomonas
uncultured Spirochaetaceae
Fructobacillus
uncultured Spirochaetaceae
Fructobacillus
Asaia
Tatumella
Acinetobacter
Zymobacter
Salmonella
Pantoea
Delftia
Clostridium sensu stricto 1
Clostridium sensu stricto 18
Burkholderia
uncultured Gammaproteobacteria
Escherichia-Shigella
Enterobacter
Pseudomonas
uncultured Cyanobacteria
Chromobacterium
Romboutsia
Serratia
Shewanella
Dysgonomonas

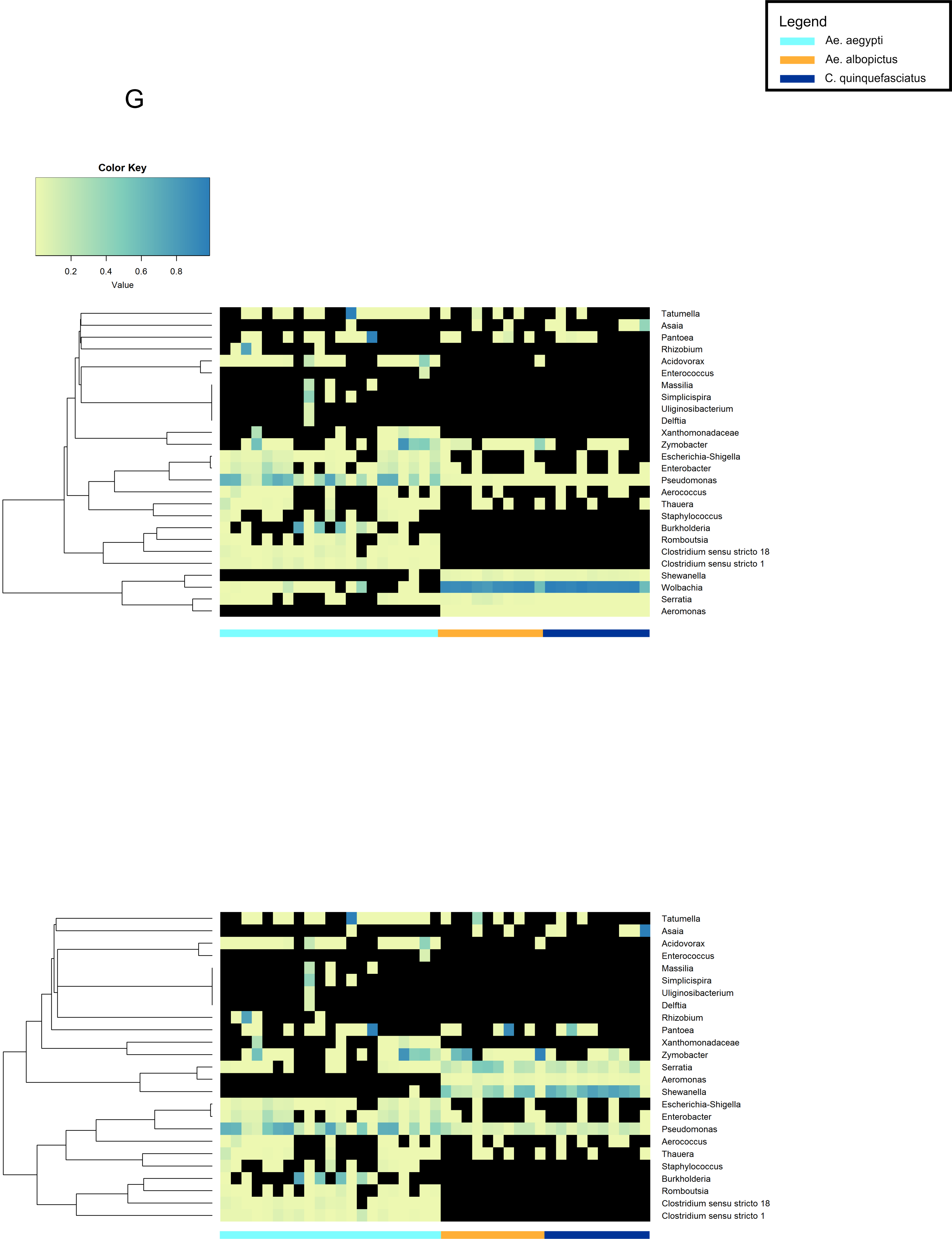

G
Legend
Ae. aegypti
Ae. albopictus
C. quinquefasciatus
Color Key
0.2
0.4
0.6
0.8
Value
Tatumella
Asaia
Pantoea
Rhizobium
Acidovorax
Enterococcus
Massilia
Simplicispira
Uliginosibacterium
Delftia
Xanthomonadaceae
Zymobacter
Escherichia-Shigella
Enterobacter
Pseudomonas
Aerococcus
Thauera
Staphylococcus
Burkholderia
Romboutsia
Clostridium sensu stricto 18
Clostridium sensu stricto 1
Shewanella
Wolbachia
Serratia
Aeromonas
Tatumella
Asaia
Acidovorax
Enterococcus
Massilia
Simplicispira
Uliginosibacterium
Delftia
Rhizobium
Pantoea
Xanthomonadaceae
Zymobacter
Serratia
Aeromonas
Shewanella
Escherichia-Shigella
Enterobacter
Pseudomonas
Aerococcus
Thauera
Staphylococcus
Burkholderia
Romboutsia
Clostridium sensu stricto 18
Clostridium sensu stricto 1

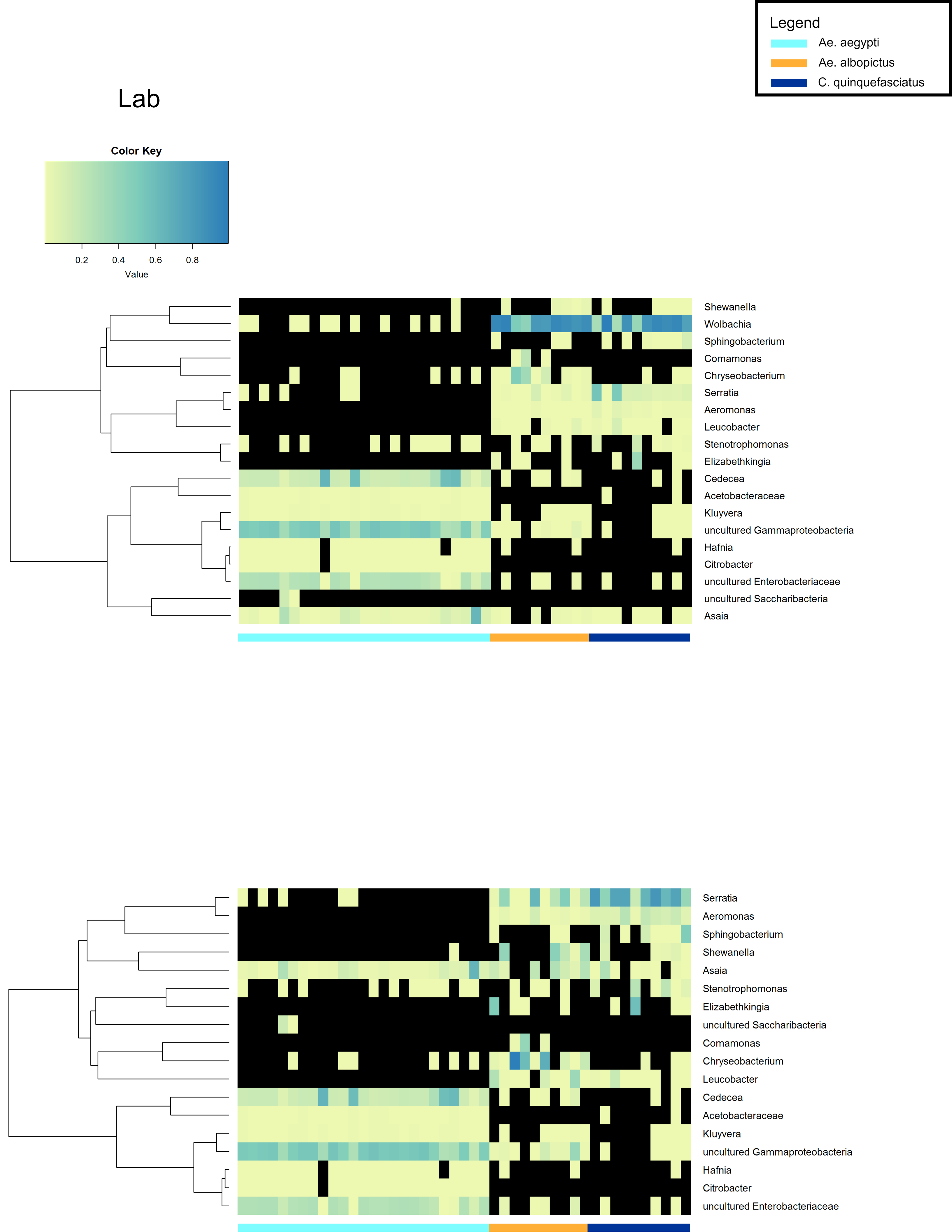
Legend
Ae. aegypti
Ae. albopictus
C. quinquefasciatus
Lab
Color Key
0.2
0.4
0.6
0.8
Value
Shewanella
Wolbachia
Sphingobacterium
Comamonas
Chryseobacterium
Serratia
Aeromonas
Leucobacter
Stenotrophomonas
Elizabethkingia
Cedecea
Acetobacteraceae
Kluyvera
uncultured Gammaproteobacteria
Hafnia
Citrobacter
uncultured Enterobacteriaceae
uncultured Saccharibacteria
Asaia
Serratia
Aeromonas
Sphingobacterium
Shewanella
Asaia
Stenotrophomonas
Elizabethkingia
uncultured Saccharibacteria
Comamonas
Chryseobacterium
Leucobacter
Cedecea
Acetobacteraceae
Kluyvera
uncultured Gammaproteobacteria
Hafnia
Citrobacter
uncultured Enterobacteriaceae

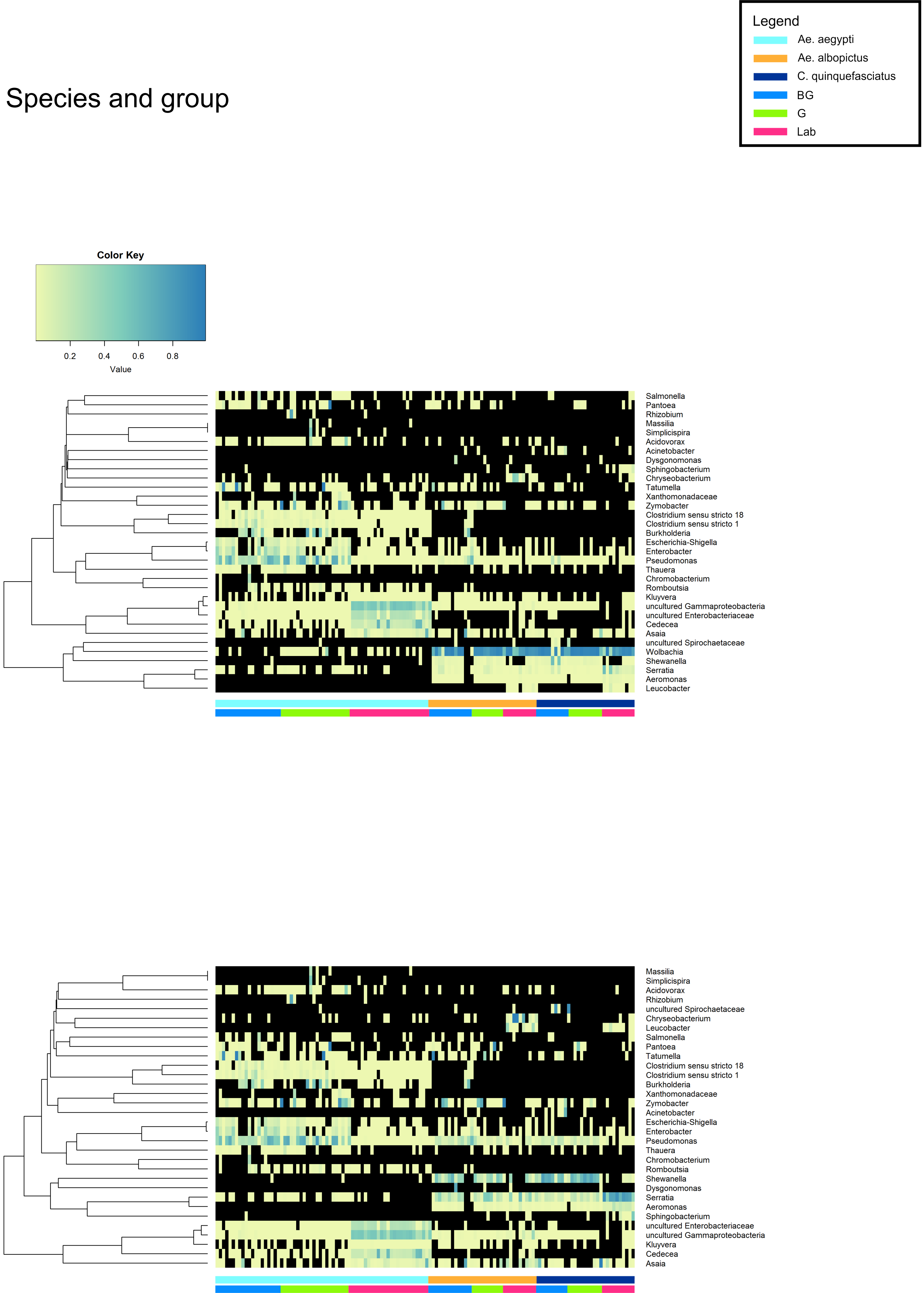
Species and group
Legend
Ae. aegypti
Ae. albopictus
C. quinquefasciatus
BG
G
Lab
Color Key
0.2
0.4
0.6
0.8
Value
Salmonella
Pantoea
Rhizobium
Massilia
Simplicispira
Acidovorax
Acinetobacter
Dysgonomonas
Sphingobacterium
Chryseobacterium
Tatumella
Xanthomonadaceae
Zymobacter
Clostridium sensu stricto 18
Clostridium sensu stricto 1
Burkholderia
Escherichia-Shigella
Enterobacter
Pseudomonas
Thauera
Chromobacterium
Romboutsia
Kluyvera
uncultured Gammaproteobacteria
uncultured Enterobacteriaceae
Cedecea
Asaia
uncultured Spirochaetaceae
Wolbachia
Shewanella
Serratia
Aeromonas
Leucobacter
Massilia
Simplicispira
Acidovorax
Rhizobium
uncultured Spirochaetaceae
Chryseobacterium
Leucobacter
Salmonella
Pantoea
Tatumella
Clostridium sensu stricto 18
Clostridium sensu stricto 1
Burkholderia
Xanthomonadaceae
Zymobacter
Acinetobacter
Escherichia-Shigella
Enterobacter
Pseudomonas
Thauera
Chromobacterium
Romboutsia
Shewanella
Dysgonomonas
Serratia
Aeromonas
Sphingobacterium
uncultured Enterobacteriaceae
uncultured Gammaproteobacteria
Kluyvera
Cedecea
Asaia
