# Supplementary material for: Microbiome Interaction Networks and Community Structure From Laboratory-Reared and Field-Collected Aedes aegypti, Aedes albopictus, and Culex quinquefasciatus Mosquito Vectors
Source: Front Microbiol. 2018 Sep 10;9:2160. doi: 10.3389/fmicb.2018.02160 (PMC6140713; doi:10.3389/fmicb.2018.02160)
Supplement: FIGURE S6 — Examples of co-occurrence and co-exclusion microbial pairs identified in the interaction networks. Scatterplots of relative abundance profiles displaying statistically significant co-occurrence and co-exclusion patterns in mosquito groups. Points represent the relative abundance values of the pair in each sample. [file Image_6.PDF]

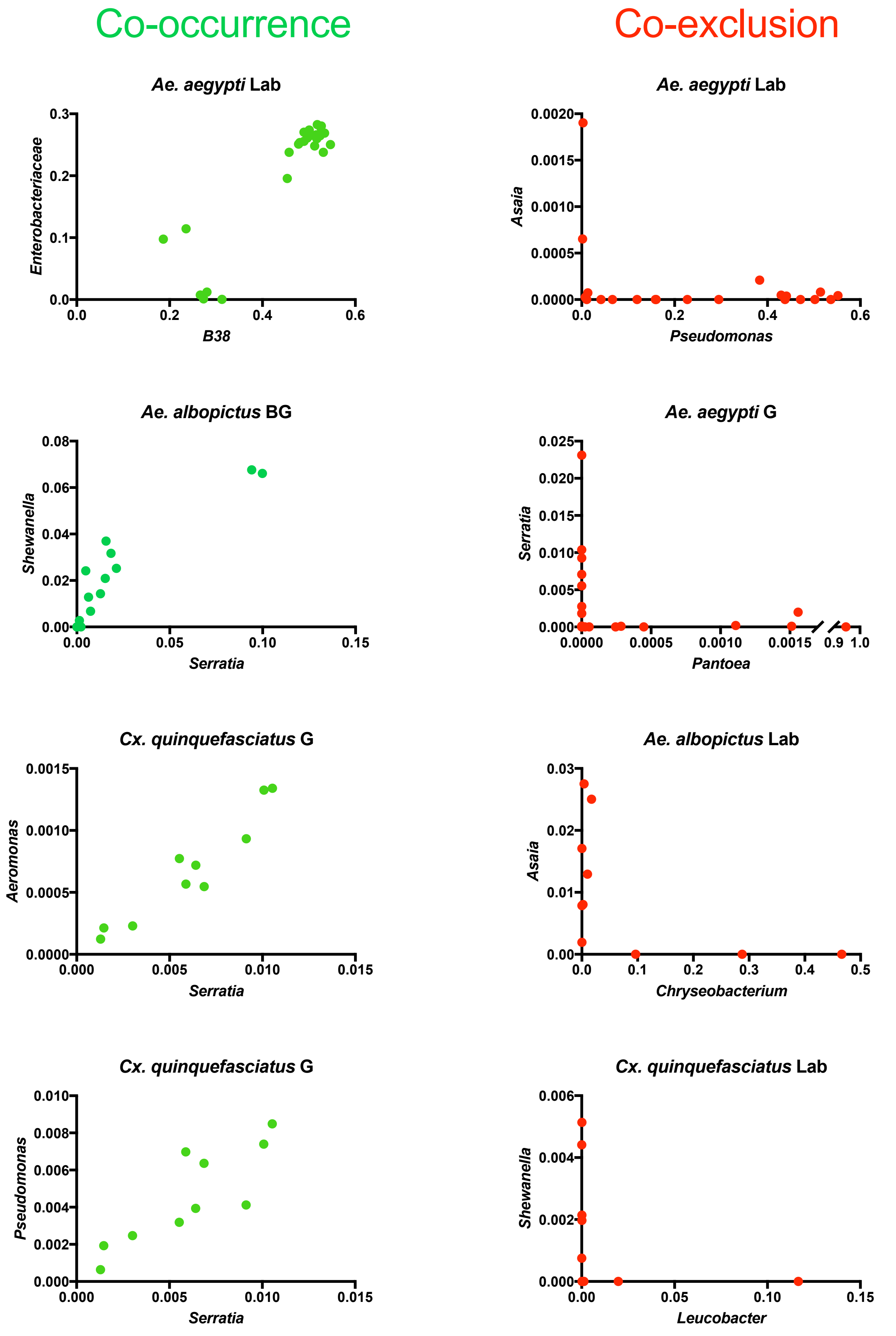
Co-occurrence
Co-exclusion
Ae. aegypti Lab
Enterobacteriaceae
B38
0.0
0.1
0.2
0.3
0.4
0.6
Ae. aegypti Lab
Asaia
Pseudomonas
0.0000
0.0005
0.0010
0.0015
0.0020
Ae. albopictus BG
Shewanella
Serratia
0.00
0.02
0.04
0.06
0.08
0.05
0.10
0.15
Ae. aegypti G
Serratia
Pantoea
0.000
0.005
0.010
0.015
0.020
0.025
0.0000
0.0005
0.0010
0.0015
0.9
1.0
Cx. quinquefasciatus G
Aeromonas
Serratia
0.0000
0.0005
0.0010
0.0015
0.000
0.005
0.010
0.015
Ae. albopictus Lab
Asaia
Chryseobacterium
0.00
0.01
0.02
0.03
0.0
0.1
0.2
0.3
0.4
0.5
Cx. quinquefasciatus G
Pseudomonas
Serratia
0.000
0.002
0.004
0.006
0.008
0.010
0.000
0.005
0.010
0.015
Cx. quinquefasciatus Lab
Shewanella
Leucobacter
0.000
0.002
0.004
0.006
0.00
0.05
0.10
0.15
